# Bone T-Scores and Functional Status: A Cross-Sectional Study on German Elderly

**DOI:** 10.1371/journal.pone.0008216

**Published:** 2009-12-09

**Authors:** Shoma Berkemeyer, Jochen Schumacher, Ulrich Thiem, Ludger Pientka

**Affiliations:** 1 Department of Geriatrics, University of Bochum, Marienhospital Herne, Herne, Germany; 2 Department of Internal Medicine, Alfried Krupp Krankenhaus Steele, University of Duisburg-Essen, Essen, Germany; National Institute on Aging, United States of America

## Abstract

**Background:**

We explore the association between bone T-scores, used in osteoporosis diagnosis, and functional status since we hypothesized that bone health can impact elderly functional status and indirectly independence.

**Methods:**

In a cross-sectional study (2005–2006) on community dwelling elderly (> = 75 years) from Herne, Germany we measured bone T-scores with Dual-energy X-ray Absorptiometry, and functional status indexed by five geriatric tests: activities of daily living, instrumental activities of daily living, test of dementia, geriatric depression score and the timed-up-and-go test, and two pooled indexes: raw and standardized. Generalized linear regression was used to determine the relationship between T-scores and functional status.

**Results:**

From 3243 addresses, only 632 (19%) completed a clinical visit, of which only 440 (male∶female, 243∶197) could be included in analysis. T-scores (−0.99, 95% confidence interval [CI], −1.1–0.9) predicted activities of daily living (95.3 CI, 94.5–96.2), instrumental activities of daily living (7.3 CI, 94.5–96.2), and timed-up-and-go test (10.7 CI, 10.0–11.3) (*P*< = 0.05). Pooled data showed that a unit improvement in T-score improved standardized pooled functional status (15 CI, 14.7–15.3) by 0.41 and the raw (99.4 CI, 97.8–101.0) by 2.27 units. These results were limited due to pooling of different scoring directions, selection bias, and a need to follow-up with evidence testing.

**Conclusions:**

T-scores associated with lower functional status in community-dwelling elderly. Regular screening of osteoporosis as a preventive strategy might help maintain life quality with aging.

## Introduction

Osteoporosis, marked by low bone density, is a common geriatric disease [1]. The NHANES Study III reported that the frequency of osteoporosis more than doubles from decade fifties to eighties [2]. Osteoporosis is clinically marked with a fracture event [3]. Risk of fractures increases with age [4]–[6]; largest cost of osteoporosis being ascribed to hip fractures [7]. Additional costs can arise due to long-term care and dependency [8]. A study reported a decline in the activities of daily living (ADL) in people with osteoporosis [9]. It appears thus that, osteoporosis could impact functional status and life-quality [10].

As like the ADL, the Timed-up-and-go test (TUG), an index for physical mobility, is yet another geriatric tool indexing functional status [11], [12]. TUG is often used to predict fractures; though specifically more often used to predict falling [13]–[14]. Since around 89% of fractures are triggered by falling [8], TUG is required to be controlled for, for outcomes like falling and fractures. Higher rate of osteoporosis has been also observed in people with lower mobility [15]. A physiological explanation could be that the loss of muscles (lower mobility), e.g., sarcopenia or weight loss, lowers bone density [16], [17]. Yet not much is known about the relationship of osteoporosis *per se* (instead of fractures/falling) and functional status.

Osteoporosis poses an existing public health challenge [7], [18], [19], which can be expected to rise, given the prognosis of a growing elderly population [20]. A political will towards a greater investment in health-care is in consideration [21] and some parts of world are grappling with huge cost of osteoporosis [7], [8] that prevention seems a suitable alternative. However, often due to the association of fractures as a clinical end-point in the practical diagnosis of osteoporosis [3], it renders the situation too late for a timely osteoporosis control.

In this article we present an analysis examining the relationship between osteoporosis and indexes of functional status. We use it to attend the debate if functional status can be regarded only as osteoporosis (disease) explanatory or can functional status be also impacted by osteoporosis (disease). The latter possibility offers caveat for multiplication of the costs of osteoporosis supporting the case for its prevention. We assumed that prevention of osteoporosis is useful not only for economic rationality but also for patient's life quality, and that community-dwelling elderly have a higher life quality and well-being than those institutionalized. We hypothesized that osteoporosis will affect functional status in elderly.

## Methods

### Ethics Statement

This study was conducted according to the principles expressed in the Declaration of Helsinki. The study was approved by the Institutional Review Board of the University of Bochum (reference number: 2383).

All patients provided written informed consent for the collection of samples and subsequent analysis.

### Subjects

**Figure 1:**
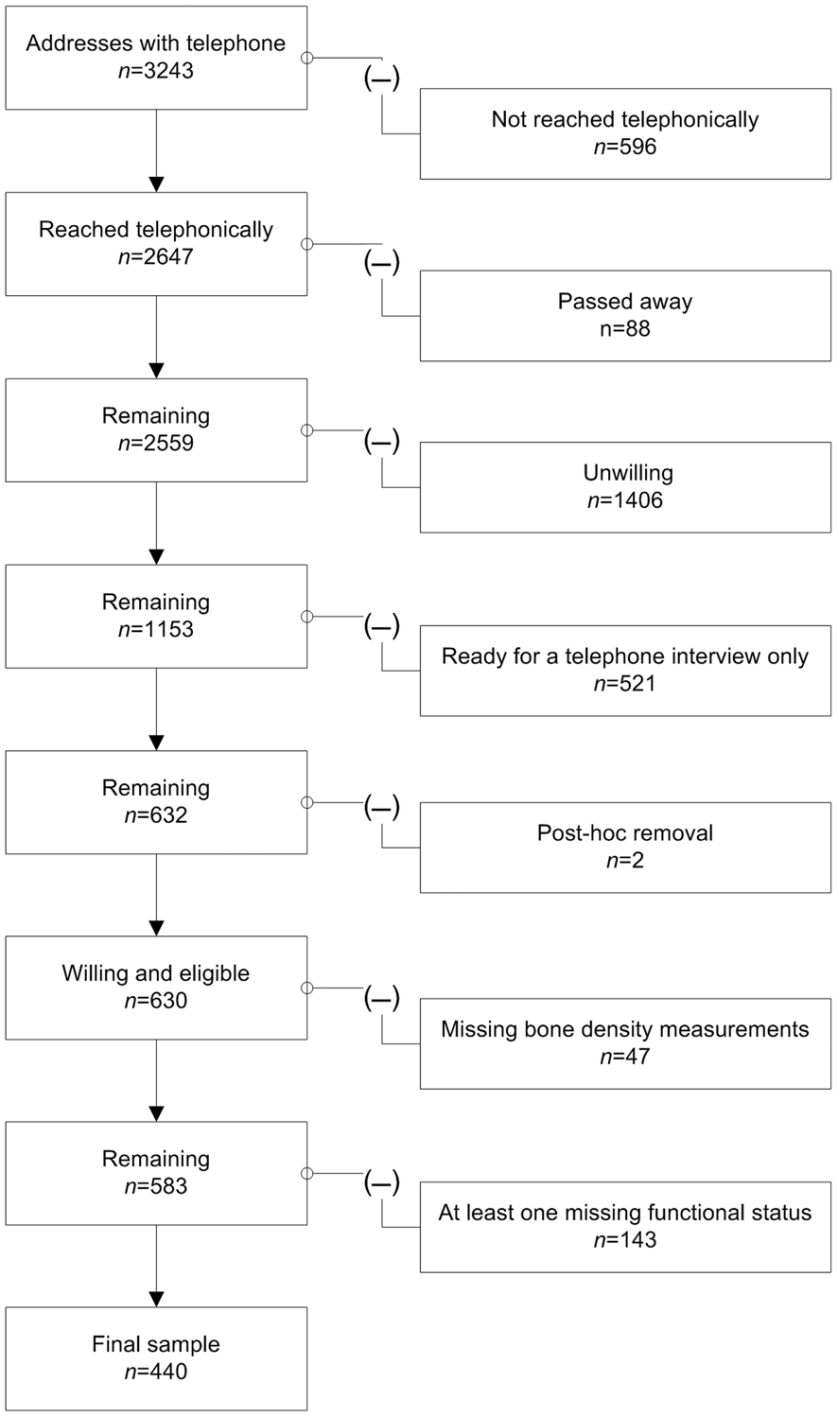
Flow-chart of study participants.

Data was collected as a cross-sectional sampling from Herne, Germany (2005–2006, available 2008) for people aged 75 years and older (+75y) who were community dwelling. They were identified using city population register (details Figure 1). From an original 3243 addresses, only 2647 could be reached telephonically. Of these only 632 came into question for the present study since the 632 completed a clinical visit. Two participants had to be excluded *post-hoc*, as they were not community dwelling, but withheld information in order to be clinically examined. Of the 630, 143 had missing values for at least one of the five functional status assessment tests and another 47 had missing bone density measurements, reducing the total working sample to 440.

Willing and eligible (age and setting criteria) participants were invited for a clinical visit at the Department of Geriatrics-Marienhospital, University of Bochum. Initially a standardized, self-administered questionnaire was filled out by the participants. This was followed by a standardized, clinical interview by study physicians. Measurements of anthropometry, bone density, and functional tests were taken by six quality-monitored personnel. All instruments were calibrated and validated before, during and after the study period. Informed written consents were obtained from all participants. Given that the participants were very elderly, we respected the participant's wish to discontinue (or inability to continue) any aspect(s) of data gathering. Data was collected July 2005 to July 2006.

### Measurements

Height was measured (0.1 cm) using Seca scale, Mod 220 (Seca Deutschland, Hamburg, Germany). Weight was measured (0.01 kg) using weighing scale WT KERN (DKD-LABOR, Balingen, Germany). Comorbidity was elicited by summing up answers from self-administered questionnaire, awarding a score of one per morbidity, so that the maximum score could be ten. The ten listed morbidities (observed most frequently in geriatric patients) were Parkinson's disease, paralysis, stroke, diabetes mellitus, hypertension, alcoholism, giddiness, gastric ulcer, osteoarthritis, and cancer.

Bone density measurements were taken at the left trochanter with a dual-energy X-ray absorptiometry (Lunar Prodigy, GE Healthcare, Munich, Germany). Osteoporosis was defined by classifying the continuous T-score measurements as per the World Health Organization (WHO) [22] (for women). It has been shown to be reasonable to adopt the same T-score cut-offs even in men [23]. Three groups were identified: osteoporosis, osteopenia, and (a presumably) healthy (bones) group.

We indexed functional status with five variables. The Barthel index of activities of daily living (ADL) [24] (modified for Germany by the Hamburg model [11], [25] and the instrumental activities of daily living (IADL) [26] were used to index gross and fine activities/disabilities, respectively. ADL awards a maximum score of 100, which indicates maximum independence. IADL is used to detect more subtle disability, which can interfere with independent functioning and awards a maximum score of 8, which indicates maximum independence. Both these were assessed as part of the clinical interview, with the each question read out to the participant.

We indexed cognition with the dementia detection test (DemTect) [27]. DemTect awards a maximum score of 18. Scores ≥13 indicate normal cognition, a score between nine and 12 indicates mild cognitive impairment, and scores below eight are classified as dementia. Geriatric depression scale (GDS) indexed psychological depression [11], [12]. A GDS score of zero to five is classified normal, six to ten light depressive, and eleven and above as heavy depression.

Mobility was indexed with the Timed-up-and-go test (TUG) [11], [28]. A chair at height 47 cm with arm-rests, two floor markings for 3 m (begin, end), and a stop-watch were used to measure TUG. The participants were told to sit down relaxed, with back at chair-end and with hands on the arm-rest. At word “go” they had to get up, walk to and return the 3 m marks on the floor and sit down again, as in the outgoing position. Stop watch was started when the participant crossed the first floor mark and stopped when crossed the second. A second reading was taken when the participant returned to sit down. The final reading was an average of the two. The test when clocked ≤10 seconds is classified normal mobility, 20 to 29 seconds as light impairment, and ≥30 seconds as heavy impairment. All data were checked for consistency before made available.

We also defined a simple summation statistic (Fscore) for functional status indexes, i.e., ADL, IADL, DemTect, GDS, and TUG, as a net index of independent gross and fine activity, cognitive and psychological well-being, and mobility. Fscore thus awarded an optimal score in the range of 111 to 141. Maximum scores of ADL (hundred), IADL (eight), and Demtect (eighteen), (higher the score, better the outcome) were added to yield an intermediate sum of 126. With GDS and TUG the scoring direction changes (higher the score, worse the outcome). Normal scores of GDS (one to five) and TUG (less than equal to 10 seconds) yielded another intermediate sum of 15. This was summed up with in126 to yield the upper end optimal Fscore of 141. The lower end optimal Fscore was given by subtracting 15 from 126; thus 111. The scoring of ADL, IADL, Demtect, and GDS are on the ordinal scale where as TUG is on the time scale. We assumed TUG to be ordinal in pooled data. Finally, we standardized the Fscore (SFscore) by taking an average of individual five scores, where each individual score was divided by the group standard deviation and then summed up.

### Statistical Rationale and Analysis

Classically geriatric assessment tools are used to assess functional abilities in the elderly. This lends to hypothesize that any geriatric disease could affect the functional abilities. We hypothesized that osteoporosis (T-scores) could associate with functional abilities, which ultimately indicate self-sufficiency, independence, and, indirectly, life quality in the elderly. Thus, our primary predictor of interest was the individual-specific T-score. To test the relationship between osteoporosis (T-scores) and functional status (ADL, IADL, DemTect, GDS, TUG, Fscore and SFscore) we used generalized linear models, which allow the flexibility of using continuous and categorical variables, and unbalanced designs. All runs were adjusted for age, sex, weight, and height (as they could confound), and comorbidity (as it could mediate). Sex was coded as 0 (female) and 1 (men). A *P* value≤0.05 was considered statistically significant. Assuming the adequate power level to be 0.80, we conducted a postanalysis power calculation (2-tailed *α* = 0.05) for our sample regressions, which yielded values exceeding 0.99 for each run. With this relatively high power we tended to accept (failed to reject) the hypothesis that osteoporosis related to functional status. Group stratified regression analysis was avoided since there would be some information loss, given that we had continuous T-score variable as predictor. All statistical analysis was carried out with SAS version 9.1 (SAS Inc., Cary, NC, USA). Group differences were conducted using ANOVA, confirmed with Tukey's test, and the Kruskal-Wallis ANOVA.

## Results

The whole sample functional status indexes showed near normal scores or a slight degree of impairment only (Table 1). Likewise, T-score statistic for the whole sample showed that on an average there was no osteoporosis across +75y olds of our sample. However, the group-stratified results indicated a significant difference in T-scores across the groups, along with significant differences in age, weight, and height (Table 2). 9.8% were with osteoporosis, 45.7% were with osteopenia, and 44.5% with healthy bones. Among the functional status indexes Demtect, Fscore, and SFscore was significantly different across the groups (Table 3). Osteoporotic group had the lowest Demtect score of 11.7 (mild cognitive impairment).

**Table 1:** Whole Sample (*n* = 440) characteristics.

Variable	Mean	95% CI
Male/Female	243/197	-
Age (years)	80.0	[79.7–80.3]
Weight^*^ (kg)	76.9	[75.7–78.1]
Height (cm)	163.6	[162.8–64.5]
T-score	−0.99	[−1.1–0.9]
Comorbidity	1.8	[1.7–2.0]
ADL	95.3	[94.5–96.2]
IADL	7.3	[7.2–7.5]
DemTect^*^	13.3	[12.9–13.6]
GDS	5.9	[5.7–6.0]
TUG	10.7	[10.0–11.3]
Fscore^*^	99.4	[97.8–101.0]
SFscore^*^	15.0	[14.7–15.3]

CI, Confidence interval; ADL, Activities of daily life; IADL, Instrumental activities of daily living; DemTect, Dementia test; GDS, Geriatric depression score; TUG, Timed up and go test; Fscore, Functional score; SFscore, Standardized functional score.

**Table 2:** Group-Stratified Characteristics.

Variable	Category	Mean	95% CI
Male/Female	Osteoporosis	16/27	-
	Osteopenia	97/104	-
	Healthy	130/66	-
Age* (years)	Osteoporosis	81.6^a,b^	[80.2–82.9]
	Osteopenia	79.9^a^	[79.5–80.4]
	Healthy	79.8^b^	[79.3–80.2]
Weight* (kg)	Osteoporosis	63.2^a,b^	[60.0–66.5]
	Osteopenia	75.0^a,c^	[73.3–76.7]
	Healthy	81.9^b,c^	[80.3–83.5]
Height* (cm)	Osteoporosis	159.3^a^	[156.3–162.4]
	Osteopenia	162.2^b^	[160.9–163.5]
	Healthy	166.0^a,b^	[164.8–167.3]
Comorbidity	Osteoporosis	1.8	[1.4–2.2]
	Osteopenia	1.8	[1.6–2.0]
	Healthy	1.9	[1.7–2.1]
T-score*	Osteoporosis	−3.06^a,b^	[−3.2–−2.9]
	Osteopenia	−1.64^a,c^	[−1.7–−1.6]
	Healthy	0.13^b,c^	[−0.03–0.3]

CI, Confidence interval.

* Significantly different between three groups (*P*≤0.05) tested with ANOVA and confirmed with Tukey's test (^a–c^).

**Table 3:** Group-Stratified Functional Status.

Variable	Category	Mean	95% CI
ADL	Osteoporosis	94.0	[91.1–96.8]
	Osteopenia	94.7	[93.3–96.2]
	Healthy	96.2	[95.2–97.2]
IADL	Osteoporosis	6.8	[6.2–7.4]
	Osteopenia	7.3	[7.1–7.5]
	Healthy	7.5	[7.3–7.7]
DemTect*	Osteoporosis	11.7	[10.5–12.9]
	Osteopenia	13.4	[12.9–13.9]
	Healthy	13.5	[13.0–14.0]
GDS	Osteoporosis	6.1	[5.6–6.6]
	Osteopenia	5.9	[5.7–6.1]
	Healthy	5.8	[5.6–6.0]
TUG	Osteoporosis	12.7	[9.2–16.2]
	Osteopenia	11.1	[10.1–12.1]
	Healthy	9.8	[9.1–10.4]
Fscore*	Osteoporosis	93.7	[86.8–100.6]
	Osteopenia	98.4	[95.8–101.0]
	Healthy	101.6	[99.9–103.4]
SFscore*	Osteoporosis	13.8	[12.7–15.0]
	Osteopenia	14.9	[14.5–15.3]
	Healthy	15.4	[15.0–15.7]

CI, Confidence interval; ADL, Activities of daily life; IADL, Instrumental activities of daily living; DemTect, Dementia test; GDS, Geriatric depression score; TUG, Timed up and go test; Fscore, Functional score; SFscore, Standardized functional score.

* Significantly different between three groups (*P*≤0.05) tested with Kruskal-Wallis ANOVA.

**Figure 2:**
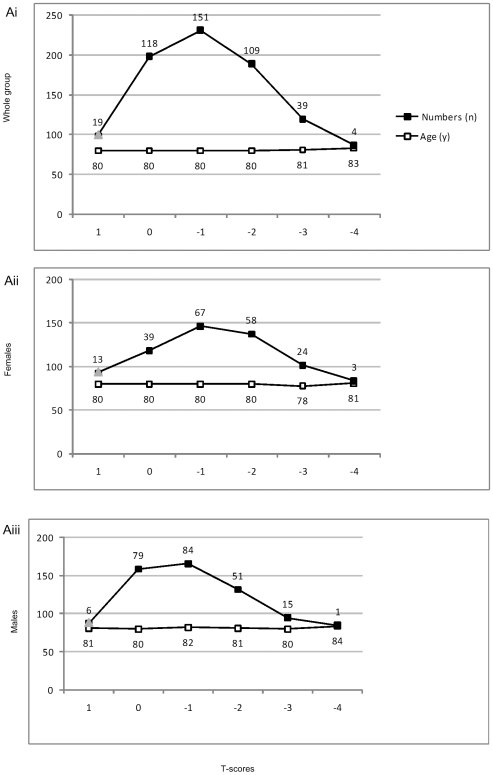
Age and numbers across T-scores for whole group (Ai), females (Aii), and males (Aiii).

Sex and group-stratified results are presented in Tables 4 and 5. Women had significantly different Demtect scores (Table 4) and men had significantly different scores in ADL, IADL, Fscore, and SFscore across groups (Table 5). Figure 2 gives the distribution of men and women across the T-scores. The women had a largely normal distribution of T-scores, at a fairly constant age of 80. The men had a right skewed distribution of T-scores, at a slight raise in age from 81 to 84. Men exceeded women in the healthy group. Women exceeded men in osteopenic and osteoporotic groups. These results confirm the analysis strategy to control for these variables in the regressions.

**Table 4:** Group-Stratified Functional Status in Females (*n* = 197).

Variable	Category	Median	Interquartile Range
ADL	Osteoporosis	100	(95–100)
	Osteopenia	100	(95–100)
	Healthy	100	(95–100)
IADL	Osteoporosis	8	(8–8)
	Osteopenia	8	(8–8)
	Healthy	8	(8–8)
DemTect*	Osteoporosis	15	(13–17)
	Osteopenia	14	(12–17)
	Healthy	13	(10–15)
GDS	Osteoporosis	5	(5–7)
	Osteopenia	6	(5–7)
	Healthy	6	(5–7)
TUG	Osteoporosis	9.1	(7.3–11.9)
	Osteopenia	9.9	(7.8–12.5)
	Healthy	9.8	(7.0–12.5)
Fscore	Osteoporosis	104.8	(98.3–110.7)
	Osteopenia	104.5	(94.7–109.2)
	Healthy	100.3	(89.3–107.4)
SFscore	Osteoporosis	16.0	(14.8–17.2)
	Osteopenia	16.0	(14.2–17.2)
	Healthy	14.9	(13.1–16.8)

CI, Confidence interval; ADL, Activities of daily life; IADL, Instrumental activities of daily living; DemTect, Dementia test; GDS, Geriatric depression score; TUG, Timed up and go test; Fscore, Functional score; SFscore, Standardized functional score.

* Significantly different between three groups (*P*≤0.05) tested with Kruskal-Wallis ANOVA.

**Table 5:** Group-Stratified Functional Status in Males (*n* = 243).

Variable	Category	Median	Interquartile Range
ADL*	Osteoporosis	100	(95–100)
	Osteopenia	100	(95–100)
	Healthy	95	(85–100)
IADL*	Osteoporosis	8	(7–8)
	Osteopenia	8	(7–8)
	Healthy	6.5	(4–8)
DemTect	Osteoporosis	14	(11–16)
	Osteopenia	13	(11–16)
	Healthy	10.5	(8–14)
GDS	Osteoporosis	6	(5–6)
	Osteopenia	6	(5–7)
	Healthy	6	(5.5–7)
TUG	Osteoporosis	8.4	(6.9–11.0)
	Osteopenia	8.5	(7.1–11.0)
	Healthy	9.2	(7.0–12.3)
Fscore*	Osteoporosis	105.2	(97.0–110.1)
	Osteopenia	104.0	(93.9–108.0)
	Healthy	99.4	(78.9–104.4)
SFscore*	Osteoporosis	15.8	(14.5–16.9)
	Osteopenia	15.2	(13.7–16.3)
	Healthy	14.2	(9.2–16.0)

CI, Confidence interval; ADL, Activities of daily life; IADL, Instrumental activities of daily living; DemTect, Dementia test; GDS, Geriatric depression score; TUG, Timed up and go test; Fscore, Functional score; SFscore, Standardized functional score.

* Significantly different between three groups (*P*≤0.05) tested with Kruskal-Wallis ANOVA.

T-scores (osteoporosis) predicted ADL, IADL, and TUG (Table 6). Demtect which showed differences across the groups (refer Table 1) was not predicted by T-scores, once other variables were controlled for. GDS indicated only a trend association (*β* = −0.10; *P* = 0.07). Pooled data showed that a decline of a unit in T-score improved SFscore by 0.41 units. This was an improvement of 2.27 units on Fscore. Given our study specific optimal range of Fscore was between 111–141 (refer Methods) and the group average Fscore was 99.4 (refer Table 1), a unit improvement in T-score could, theoretically, improve the Fscore from the group average of 99.4 to 101.67.

**Table 6:** Relationship of T-scores with Functional Status.

Outcome	Explanatory	Parameter Value	S.E.	*p*-Value	Model *R^2^*
ADL	T-scores	1.07	0.37	0.0041	
	Age	0.05	0.005	<0.0001	
	Comorbidity	−1.92	0.30	<0.0001	
	Weight	−0.11	0.04	0.0056	0.13
IADL	T-scores	0.20	0.06	0.0005	
	Age	−0.07	0.02	0.0003	
	Comorbidity	−0.13	0.05	0.0079	
	Sex	−0.48	0.19	0.0114	0.10
DemTect	T-scores	0.19	0.15	0.1887	
	Age	−0.33	0.04	<0.0001	
	Sex	−1.81	0.47	0.0002	
	Height	0.06	0.03	0.0142	0.14
GDS	T-scores	−0.10	0.06	0.07	
	Age	0.05	0.02	0.0089	
	Comorbidity	0.30	0.05	<0.0001	0.11
TUG	T-scores	−0.71	0.27	0.0086	
	Age	0.43	0.09	<0.0001	
	Comorbidity	1.71	0.22	<0.0001	
	Weight	0.10	0.03	0.0004	0.20
Fscore	T-scores	2.27	0.65	0.0006	
	Age	−1.22	0.22	<0.0001	
	Comorbidity	−4.18	0.53	<0.0001	
	Weight	−0.23	0.07	0.001	0.22
SFscore	T-scores	0.41	0.11	0.0004	
	Age	−0.23	0.04	<0.0001	
	Comorbidity	−0.60	0.09	<0.0001	
	Weight	−0.03	0.01	0.0298	
	Sex	−1.00	0.37	0.0075	0.20

S.E., Standard Error; ADL, Activities of daily life; IADL, Instrumental activities of daily living; DemTect, Dementia test; GDS, Geriatric depression score; TUG, Timed up and go test; Fscore, Functional score; SFscore, Standardized functional score.

## Discussion

We explained functional status (ADL, IADL, Demtect, GDS, and TUG) with T-scores and report T-scores predicted ADL, IADL and TUG, suggesting that impact of osteoporosis was predominantly in the domains of activity and mobility. Although cognition (Demtect) was observed to be lowest in the osteoporotic group, T-scores did not predict Demtect. The relationship of depression (GDS) with osteoporosis was also unclear.

Our hypothesized direction of causality was that osteoporosis could impact functional scores. Both Demtect (dementia) and TUG (mobility) have been used to explain osteoporotic fractures (falling). Dementia is an accepted risk factor for osteoporotic fractures [3], [29], [30]. This is supported with study results where osteoporosis has been observed in patients with cognitive impairment [30]. However, no clear report on causality in any of the two diseases occurrence has been reported [29]. In the case of TUG (mobility), literature reports its use as an explanatory for falling [14], [31], though a study could not report any clinical relevance simultaneously indicating elderly balance was more important [13]. Falling is multifactorial; one explanatory could be bone loss [16]. This could result in fractures, also osteoporotic fractures. The direction of causality would run from bone health (osteoporosis) to falling and fractures, which would also impact future mobility, even so outgoing mobility would be a criterion required to be controlled for. A reverse causality could also run at times, but not typically in geriatric patients.

It is also known that osteoporotic fractures constitute only 30% of the total fractures [1] so caution is required in extrapolating WHO's fracture risk assessment tool (FRAX) as an osteoporosis diagnosis tool [32], even so it is ideal for outcome fractures. FRAX risk diseases include not only osteoporosis but also osteoarthritis, which's etiology, is quite different from osteoporosis. Hence, it remains plausible to hypothesize that functional status, e.g., mobility, activities of life, mental health, etc., can also be predicted by osteoporosis. In this explanation osteoporotic fracture would not serve as outcome, but mediate, since osteoporosis is possible even without any fractures [3], [7].

Our results on ADL are supported by some studies which also predicted ADL with osteoporosis [8], [9], [33]. We could not support the cognitive impact (Demtect) of osteoporosis even so literature reports that dementia (also Alzheimer's) could be associated with osteoporosis [29]. As an explanation, our sampling of community-dwelling elderly eliminated institutionalized elderly, who would be affected in greater numbers and severity with both osteoporosis and dementia. Falling [34] and fractures [35]–[36] have been also associated with depression, though latter studies [35]–[36] reported no significant association. We also report primarily a not significant association. Where we distinguish is that we hypothesized psychological depression as an osteoporotic impact. Our general direction of analysis, where we predict functional status with T-scores (osteoporosis), is also supported by results of Russell and colleagues [31], who predicted functional status with fractures. The study diverges from ours in predicting functional status also with depression and TUG. We, instead, index functional status with depression (psychological domain) and TUG (mobility domain). However, longitudinal studies are required with baseline and end of study measurements to better investigate this relationship.

Our pooled results suggest that a unit improvement in T-score, i.e., a rise in our whole group average from observed −0.99 to 1.99, associated with an increase of 2.27 units on the pooled Fscore, i.e., a rise from observed 99.4 to 101.7. Our study specific optimal Fscore range was identified between 111 and 141. To that extent a strategy to prevent osteoporosis would only partially improve the composite functional status, indicating factors other than alleviation of osteoporosis are also important in defining well-being and independence in older people.

In our sample more men were without osteoporosis compared to women (men∶women, 130∶66). Almost an equal number of men and women were osteopenic (men∶women, 97∶104), and almost double the number of women had osteoporosis compared to men (men∶women, 16∶27). We also observed a greater decline in functional status in men than women (refer Tables 4 and 5). While we cannot rule out a random occurrence, an alternate could be that the spread of osteoporosis is greater in women (issue of quantity) and the impact of osteoporosis more severe in men (issue of quality), which though requires research. This is so as our study has some major limitations.

Our study could not avoid an unforeseen selection bias towards males, even so the number of women exceed men in this population segment. Further, women are known to be more health-conscious than men, so it is quite possible that the men who agreed to participate in the study were more severe cases of osteoporosis compared to women. The study further lacks in being a one-time cross-sectional analysis and hence all our results are open to evidence testing. With longitudinal data it would have been possible to control for outgoing functional status to predict end of study functional status for a more true depiction of disease impact. Also, composite functional status (Fscore), as defined by us, is highly limited in application. It is principally restricted by the inherent weakness of pooling two scoring directions, i.e., higher is better (ADL; IADL, and Demtect), and higher is worse (GDS, and TUG). We have tried to ameliorate this inadequacy by defining an optimal range. In defense, we suggest that this inadequacy in pooling was uniformly applicable to every observation in our sample. Finally, our study is also limited in representing the true impact of the disease osteoporosis due to an out-going selection of community dwelling elderly.

In summary, our results indicate that the WHO determined T-score [22] to classify osteoporosis associated with lower functional status in our community-dwelling elderly. Our analysis was unable to report association between T-scores, and cognitive dementia and psychological depression in this sample. Perhaps the most immediate impacts of osteoporosis are in the activity (ADL and IADL), and mobility (TUG) domains. We surmise that prevention of osteoporosis could help to limit this decline in functional status, in order to promote greater independence in elderly. Our findings should not be interpreted, however, as evidence providing or population representative. Future work should seek to establish the outgoing hypothesis of our study that osteoporosis can impact functional status. An inquiry into the additional geriatric diseases affecting functional status is also warranted as the population becomes increasingly older.
